# SOD3 Is a Non-Mutagenic Growth Regulator Affecting Cell Migration and Proliferation Signal Transduction

**DOI:** 10.3390/antiox10050635

**Published:** 2021-04-21

**Authors:** Alessia Parascandolo, Mikko O. Laukkanen

**Affiliations:** Center for Experimental Endocrinology and Oncology (IEOS), CNR, Via Pansini 5, 80131 Naples, Italy; al.parascandolo@libero.it

**Keywords:** extracellular superoxide dismutase, sod3, signal transduction, cancer, proliferation, migration

## Abstract

Superoxide dismutase (SOD) family isoenzymes, SOD1, SOD2, and SOD3, synthesize hydrogen peroxide (H_2_O_2_), which regulates the signal transduction. H_2_O_2_ is a second messenger able to enter into the cells through aquaporin 3 cell membrane channels and to modify protein tyrosine phosphatase activity. SOD3 has been shown to activate signaling pathways in tissue injuries, inflammation, and cancer models. Similar to the H_2_O_2_ response in the cells, the cellular response of SOD3 is dose-dependent; even a short supraphysiological concentration reduces the cell survival and activates the growth arrest and apoptotic signaling, whereas the physiological SOD3 levels support its growth and survival. In the current work, we studied the signaling networks stimulated by SOD3 overexpression demonstrating a high diversity in the activation of signaling cascades. The results obtained suggest that SOD3, although inducing cell growth and affecting various biological processes, does not cause detectable long-term DNA aberrations. Therefore, according to the present data, SOD3 is not a mutagen. Additionally, we compared SOD3-driven immortalized mouse embryonic fibroblasts to SV40 immortalized NIH3T3 cells, demonstrating a marked difference in the activation of cellular kinases. The data presented may contain important druggable targets to abrogate unwanted cell growth.

## 1. Introduction

Reactive oxygen species (ROS) are involved in the regulation of a vast number of physiological processes as a result of the aerobic metabolism of organisms. Mechanistically, the effect of ROS is channeled through a redox signaling network consisting of the main signaling cascades downstream of tyrosine kinase receptors (RTKs), small GTPases, and large G protein-coupled receptors (GPCRs), which maintain the redox balance and regulate oxidative stress [1,2,3].

Hydrogen peroxide (H_2_O_2_) is a well-characterized ROS produced by several redox enzymes. H_2_O_2_, which can enter into the cells via aquaporin cell membrane channels [4], can inactivate protein tyrosine phosphatases (PTPs) [5] by oxidizing the reactive cysteines in the active site, thereby allowing the phosphorylation of the cellular kinases at tyrosine residues [6,7] with the subsequent activation of mitogen, growth, and survival signaling. Alternatively, H_2_O_2_ can activate directly the kinases by reacting with the cysteine residues in the cytoplasmic domain of the tyrosine kinase receptors [8]. The PTP superfamily contains over 100 members that control a high number of kinases and tyrosine kinase receptors, which are potentially regulated by H_2_O_2_ [9]. Accordingly, SOD3, an extracellular copper and zinc-containing enzyme converting superoxide anion (O_2_^●−^) to H_2_O_2_, has not been shown to associate with any specific signaling cascade. The enzyme responds to local oxidative stress balancing the substrate and the dismutation reaction end product concentrations in different pathologies [10].

Correspondingly, the mRNA synthesis of *a* is regulated by an interacting network of signaling molecules, in which the activation status in individual persons correlates to age and pathology. The gene expression analysis in thyroid and colon tumors demonstrates a patient-specific *SOD3* production that can alter markedly between the patients [2]. Interestingly, recent reports suggest a tumor-supporting role for SOD1, SOD2, and SOD3 [11,12,13,14,15], demonstrating a role for them as biomarkers in tumorigenesis. This further highlights a need to investigate the function of the enzymes in different model systems [16]. Importantly, unlike in tumors in which the *SOD3* expression vary, the enzyme expression is frequently downregulated in single cancer cell cultures correlating with the activation level of the RAS oncogene and epigenetic status of the cells [17,18,19], therefore compromising the use of single-cell lines as model systems to recapitulate the overall function of SOD3 in pathologies. In vitro models, however, can be used to probe the signaling network regulated by SOD3. Therefore, to dissect the effect of SOD3 on the activation of individual signaling molecules, in the current work, we utilized different cell lines overexpressing SOD3.

## 2. Methods

### 2.1. Cell Cultures

Primary bone marrow mesenchymal stem/stromal cells (MSCs) [20], mouse embryonic fibroblasts (MEF) [21], and NIH3T3 (ATCC, Manassas, VA, USA) cells were grown in αMEM (Euroclone, Milano, Italy) supplemented with 10% defined FBS (GE-Healthcare, Chicago, IL, USA), nonessential amino acids (Euroclone), L-alanine-L-glutamine (Euroclone), and penicillin/streptomycin (Euroclone). The MSC passage count was performed using the formula (LOG10 (final number of cells/number of seeded cells)/LOG2 + previous passage number). The TPC1 papillary thyroid cancer cells were grown in DMEM 10% FBS, penicillin, and streptomycin (Euroclone). The 8505c anaplastic thyroid cancer cells (DSMZ; German collection of microorganism and cell cultures, Braunschweig, Germany) were cultured in RPMI medium (Euroclone) supplemented with 10% FBS, penicillin, and streptomycin. 

Human MSCs were transduced with MOI 2, 5, and 10 SOD3 or control lentivirus (ABM, Richmond, BC, Canada). MSCs were used for cell passage analysis, DNA correction enzyme expression analysis, and DNA mutation analysis. MEF clones were used for *sod3* expression analysis, Western blot signaling analysis, protoarray analysis, and *tgfβ* mRNA expression analysis. MEF clones were created by transduction of the cells with *gfp* or *sod3* ecotropic retrovirus MOI 10 [21]. Each MEF clone is derived from a different transduction experiment and, therefore, has a slightly different expression level of the *sod3* retrovirus. NIH3T3 cells were used in the protoarray analysis. TPC1 and 8505c cell lines stably expressing *SOD3* or the control plasmid (0.5 μg) were made by nucleofection, which creates a mixed population of cells containing different SOD3 expression levels. Three days after the transfection, Neomycin (Euroclone) was added to the cells. The long-term culture of the transfected cells in the presence of Neomycin erased non-transfected cells and cells that contained supra-physiological SOD3 concentrations. 8505c and TPC1 cells were used for the gene ontology enrichment analysis and visualization (Gorilla) analysis [21,22]. TPC1 cells were used for the migration analysis.

### 2.2. Mutation Analysis

The analysis of 8-oxo-deoxyguanosine was done using an Oxidative Damage kit according to the manufacturer’s instructions (Cayman Chemicals, Ann Arbor, MI, USA). Microsatellite analysis was done using a commercial service (BMR Genomics, Padova, Italy).

### 2.3. BrdU DNA Replication Analysis

To analyze the effect of SOD3 overexpression on MEF proliferation, MEF GFP, MEF SOD3 cl6, cl8, and cl5 were seeded on the coverslips (Waldemar Knittel Glasbearbeitungs-GmbH, Braunschweig, Germany) and grown to 60–70% confluence; after which, bromodeoxyuridine (10 mM) (BrdU) (Roche, Basel, Switzerland) was added to the culture medium for 15 min. The coverslips were fixed with an ethanol fix solution, and the BrdU-positive cells were labeled using FITC-conjugated secondary antibodies (Jackson ImmunoResearch Laboratories Inc., West Grove, PA, USA). The nuclei were counter-stained with Hoechst (Sigma, St. Louis, MO, USA). The BrdU-positive cells were counted from the high-power microscope fields.

### 2.4. Gene Expression Analysis

The mRNA was isolated from cells using the RNeasy mini kit (Qiagen, Hilden, Germany) and reverse-transcribed to cDNA by QuantiTect reverse transcription (Qiagen). SYBR Green PCR master mix (Applied Biosystems, Foster City, CA, USA) was used for qPCR. The primers are shown in Supplemental Table S1. Gene ontology enrichment analysis and visualization (Gorilla) analysis were done as described previously [22].

### 2.5. Protoarray Analysis

The cells were incubated for 24 h in serum-free medium; harvested into lysis buffer (50-mM HEPES, pH 7.5, 150-mM NaCl, 10% glycerol, 1% Triton X-100, 1-mM EGTA, 1.5-mM MgCl2, 10-mM NaF, 10-mM sodium pyro-phosphate, 1-mM Na3VO4, 10-μg aprotinin/mL, and 10-μg leupeptin/mL); and centrifuged at 10,000× *g* at 4 °C. The array was performed according to the manufacturer’s instructions (R&D Systems, Minneapolis, MN, USA).

### 2.6. Western Blot Analysis

The proteins isolated from cells were separated in SDS gel electrophoresis and transferred to Hybond C Extra nitrocellulose membranes (GE Healthcare, Chicago, IL, USA). The membranes were incubated in 5% nonfat dry milk for 1 h at room temperature and overnight at 4 °C with the primary antibody. Antibodies were p-ATM (Millipore, Darmstadt, Germany), p-Chk1 (Cell Signaling, Danvers, MA, USA), p-p53 (Cell Signaling), p21 (Cell Signaling), p-EphA2 (Cell Signaling), p-Src (Cell Signaling), p-JNK (Cell Signaling), JNK (Cell Signaling), p-cJun (Cell Signaling), PKA substrates (Cell Signaling), catalytic PKA unit (Cell Signaling), p-AKT (Cell Signaling), AKT (Cell Signaling), p-GSK3β (Cell Signaling), p-CREB (Cell Signaling), p-SMAD2/3 (Cell Signaling), SMAD 2/3 (Cell Signaling), cyclin D1 (Cell Signaling), and tubulin (Cell Signaling).

### 2.7. Cell Migration Analysis

For the migration assay, 100 μL of Matrigel (Corning Inc., Corning, NY, USA) at 1 mg/mL was added to a migration chamber (8 microns; BD, San Jose, CA, USA). The chambers were incubated at room temperature for 30 min to stabilize the Matrigel. The chambers were then moved into 12-well plates, and 50,000 TPC1 cells were added to the Matrigel. The cells were allowed to migrate for 24 h at 37 °C; after which, the Matrigel was removed. Migrated cells were fixed with 7% paraformaldehyde (Sigma), washed with PBS, and stained with crystal violet (Sigma). The number of migrated cells was counted from the microscope high-power fields.

### 2.8. Statistical Analysis

The *p*-values (* *p* < 0.05, ** *p* < 0.01, and *** *p* < 0.001) were determined by two-tail independent sample *t*-tests. The results are expressed as the mean ± SD.

## 3. Results

### 3.1. SOD3 Induced Short-Term DNA Damages

**Figure 1:**
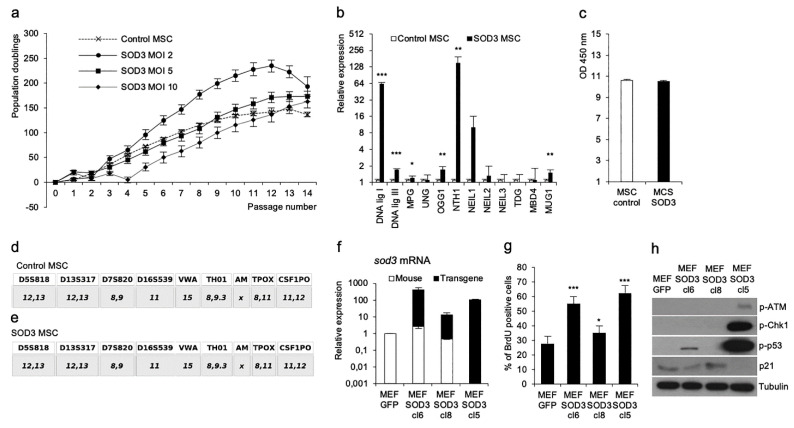
SOD3 stimulates growth without causing long-term DNA aberrations. (**a**) Dose-dependent effect of SOD3 on human MSC growth. Passage number analysis demonstrated increased cell proliferation at low MOI and decreased growth at high MOI. (**b**) DNA damage correction enzyme expression analysis of GFP and SOD3 MSCs from passage 1. The analysis demonstrated a significantly increased expression of *DNA ligase I* (*p* < 0.001), *DNA ligase III* (*p* < 0.001), *OGG1* (*p* < 0.01), *NTH1* (*p* < 0.01), and *MUG1* (*p* < 0.01). (**c**) The 8-oxo-dG analysis of GFP and SOD3 MSCs from passage 8 showed an equal presence of the modified guanosine in DNA. (**d**,**e**) STR analysis of GFP and SOD3 MSCs from passage 8 did not indicate SOD3-driven microsatellite instability. (**f**) Gene expression analysis for endogenous and transgene *sod3* mRNA synthesis. The data demonstrated variable transgene expression levels. Note, *sod3* mRNA production in MEF SOD3 cl5 was almost solely result of transgene transcription. (**g**) BrdU DNA incorporation analysis for DNA replication suggested significantly increased cell proliferation for MEF SOD3 cl6 (*p* < 0.001), MEF SOD3 cl8 (*p* < 0.05), and MEF SOD3 cl5 (*p* < 0.001), as compared to MEF GFP controls. (**h**) Western blot analysis for the in vitro MEF model composed of MEF GFP and MEF SOD3 clones 6, 8, and 5. DDR pathway activation occurred in MEF SOD3 cl5 simultaneously with the downregulation of p21. MEF SOD3 cl6 demonstrated the mild activation of p53 and moderate downregulation of p21. The *p*-values are (* *p* < 0.05, ** *p* < 0.01, and *** *p* < 0.001).

The SOD3 expression in vitro and in vivo has a dose-dependent effect; a high enzyme expression caused by the transfection of a high plasmid concentration or by adenovirus activates an immediate growth arrest in cells that have a wild-type p53 and delays the decreased growth in cells in which p53 is mutated [21,23,24,25]. We did a dose escalation for the effect of SOD3 on human MSCs by transducing the cells with *SOD3* lentivirus MOI 2, MOI 5, and MOI 10 (Figure 1a). The lowest virus dose, MOI 2, had the strongest growth stimulation effect, whereas MOI 10 markedly reduced the cell proliferation. Although the overexpression of *SOD3* stimulated the primary cell proliferation, it did not immortalize human MSCs. Instead, we observed a decline in the population doublings initiating at passage 12 (Figure 1a).

Our previous data demonstrated the activation of the DNA damage response (DDR) and increased γH2AX phosphorylation in TPC1 papillary thyroid cancer cells, suggesting DNA damage [21]. In the current work, the overexpression of *SOD3* MOI 2 in MSCs increased the DNA damage correction enzyme expression at passage 1 (Figure 1b). However, when analyzing the presence of the 8-oxo-2’-deoxyguanosine (8-oxo-dG) mutation at passage 8, we observed no difference between the control and *SOD3* transduced MSCs (Figure 1c). Additionally, the microsatellite (STR) analysis at passage 8 showed the same STR pattern in *GFP* and *SOD3*-transduced MSCs (Figure 1d,e) suggesting that the enzyme does not induce long-term DNA aberrations.

To study the effect of SOD3 in mouse embryonic primary fibroblasts, we used MEF SOD3 cl6, MEF SOD3 cl8, and MEF SOD3 cl5 cell models, which each had different expression levels of transgene rabbit *sod3* and endogenous mouse *sod3* (Figure 1f). In MEF SOD3 cl6 and cl8, the majority of the *sod3* mRNA originated from the endogenous gene, whereas, in MEF SOD3 cl5, the enzyme was synthesized solely from the transduced rabbit cDNA. Consequently, the growth modalities varied between the clones (Figure 1g). MEF SOD3 cl6 and cl5 had the highest DNA replication rate as compared to the MEF GFP control cells. In MEF SOD3 cl8, the cell proliferation was only modestly increased. The Western blot analysis demonstrated that *sod3* expression in MEF SOD3 cl5 induced the phosphorylation of ATM, CHK1, and the p53 DNA DDR pathway (Figure 1h) and downregulated p21, thereby interrupting the growth arrest of the cells. The p53 activation was modestly upregulated in MEF SOD3 cl6, correlating to a modest downregulation of p21, indicating an association of SOD3-derived p21 downregulation and increased cell proliferation. Therefore, the current and the previous data show that SOD3 overexpression alone does not initiate a malignant transformation of cells [21] and suggest that SOD3, or the reaction end product H_2_O_2_, are not carcinogens or mutagens but, rather, regulate cell proliferation.

### 3.2. SOD3 Affected a Large Variety of Cellular Functions

**Figure 2:**
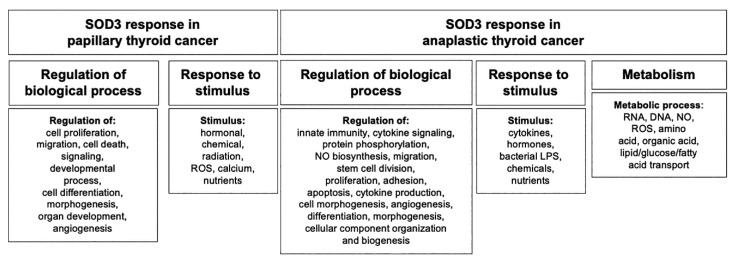
Gene ontology enrichment analysis. The analysis indicated the cancer type specificity for SOD3-driven regulation of the biological pathways. TPC1 cells model papillary thyroid cancer and 8505c cells model anaplastic thyroid cancer. SOD3 affected the expression of a vast number of genes committed to the regulation of biological pathways.

Wild-type p53 containing papillary thyroid cancer TPC1 cells and mutated p53 containing anaplastic thyroid cancer 8505c cells are commonly used to study the growth characteristics of thyroid cancer [21,22,26,27]. The analysis of gene ontology enrichment in thyroid cells overexpressing *SOD3* demonstrated a large number of biological pathways affected by the enzyme (Figure 2, Supplemental Figures S1 and S2). Interestingly, the range of the pathways affected was larger in anaplastic thyroid cancer 8505c cells than in papillary thyroid cancer TPC1 cells. Most prominently, SOD3 affected the metabolic processes in the anaplastic cancer model.

### 3.3. Activation of RTKs and Cellular Kinases

**Figure 3:**
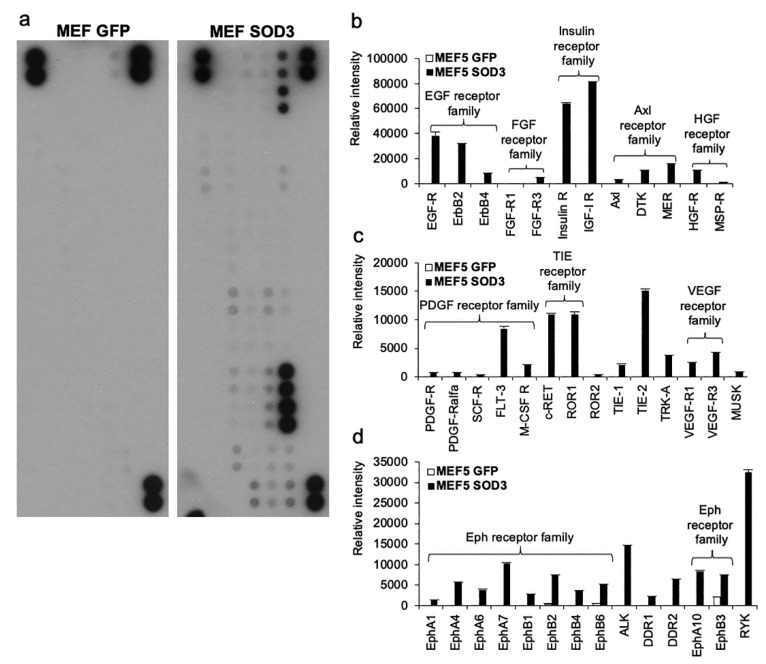
Tyrosine kinase receptor activation analysis. (**a**). MEF GFP and MEF SOD3 protoarray membranes. (**b–d**). Intensity measurements for positive signals in protoarray membranes. The analysis demonstrated EGF, insulin, AXL, HGF, PDGF, TIE, VEGF, and Eph receptor family activation by SOD3.

H_2_O_2_ can induce the temporal reorganization of the three-dimensional structure of PTPs regulating RTK phosphorylation and the activation of downstream signaling. The protoarray data of MEF GFP and MEF SOD3 cl5 showed the increased activation of several RTK families (EGF, FGF, Axl, HGF, PDGF, TIE, VEGF, Eph, and insulin) (Figure 3a–d and Supplemental Figure S3a,b), which promote growth, angiogenesis, migration, and cellular metabolism. The ability of SOD3 to stimulate the insulin receptor activation is in line with the Gorilla analysis demonstrating a marked enrichment of metabolism-related genes in anaplastic thyroid cancer cells (Supplemental Figure S2).

**Figure 4:**
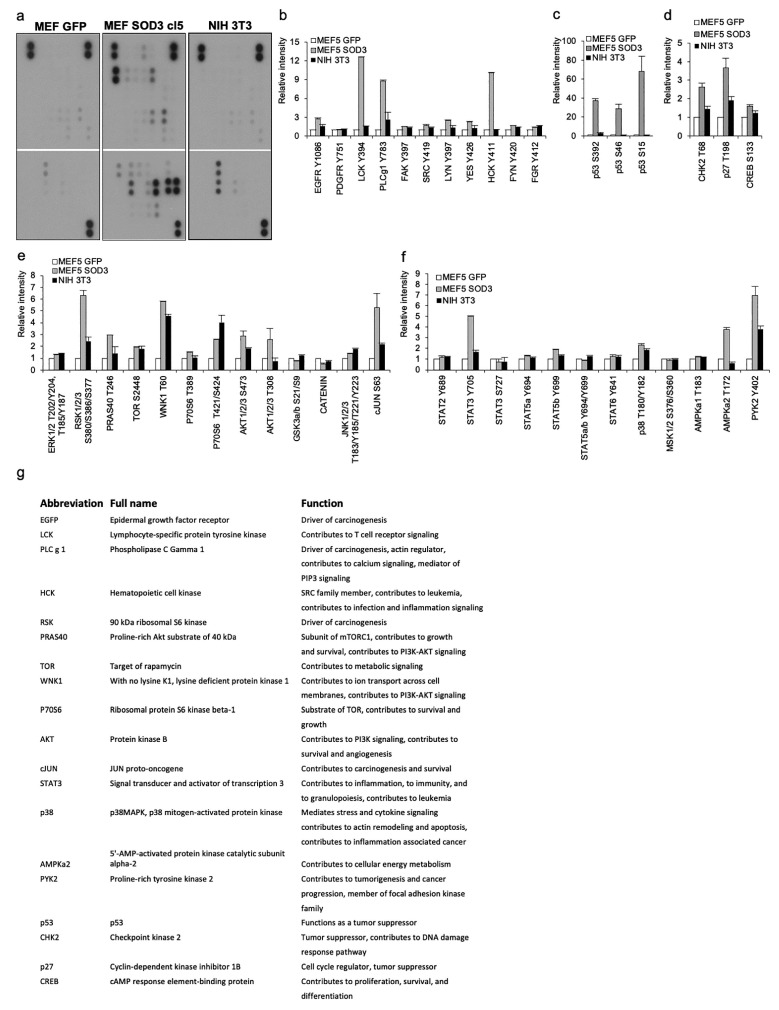
Kinase activation analysis. (**a**) The comparison of MEF GFP, MEF SOD3, and NIH3T3 demonstrated marked differences in the signaling pathways, inducing immortalization by *sod3* and *SV40*. (**b**–**f**) Groups of cell membrane-associated signaling molecules activated by SOD3. (**g**) Table showing the function of the selected signaling molecules with robust activation.

To further characterize the activation of signaling induced by SOD3, we executed a kinase protoarray (Figure 4a–f and Supplemental Figure S4a–c). SRC family proto-oncogenes LCK, FAK, SRC, LYN, YES, and FYN demonstrated increased activation, together with downstream growth-related kinases RSK1/2/3, PRAS40, WNK1, P70S6, AKT, and cJUN, at the presence of SOD3. From STAT family kinases, STAT3 showed the most prominent activation. Although several growth-related kinases were phosphorylated, we also observed the activation of the DNA damage response pathway molecules CHK2 and p53 (S15, S46, and S392). To further dissect the immortalization signaling, we compared SOD3-transduced MEF cl5 cells to SV40 immortalized NIH3T3 cells. The array suggested a markedly more narrow-scale activation of kinases by SV40 showing robust phosphorylation only for STAT3 and WNK1 (Figure 4a–f and Supplemental Figure S4a–c). The data demonstrated the difference between virus-derived and the ROS-derived immortalization of murine cells. The most obvious difference was the high phosphorylation of p53 by SOD3. SV40 achieves immortalization through Large-T antigen that binds to the p53 protein, preventing the association of the tumor suppressor to target promoters. Another crucial mechanism is the regulation of telomere lengths by viral genes to escape the Hayflick limit that controls cellular aging [26]. The data further indicated a plethora of intercellular signals induced by SOD3, suggesting a more complex nature of ROS contribution to tumorigenesis as compared to exogenous biological carcinogens, such as viruses.

**Figure 5:**
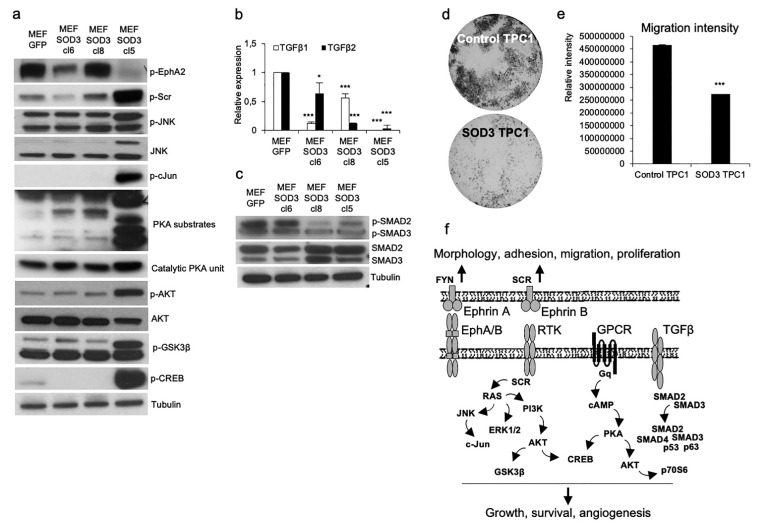
Activation analysis of specific kinases. (**a**) MEF SOD3 cl5 demonstrated the highest phosphorylation of SRC, cJUN, AKT, and CREB. In addition, we observed a markedly increased activation of PKA substrates and decrease activation of EphA2 in cl5. (**b**) The *tgfβ* mRNA analysis demonstrated significantly decreased *tgfβ1* and *tgfβ2* expression in MEF SOD3 cl6 (*p* < 0.001 and *p* < 0.05, respectively), MEF SOD3 cl8 (*p* < 0.001 and *p* < 0.001, respectively), and MEF SOD3 cl5 (*p* < 0.001 and *p* < 0.001, respectively). (**c**) Western blot analysis for the TGFβ downstream kinases SMAD2 and SMAD3. Phosphorylation of SMAD2 and SMAD3 was decreased in MEF SOD3 cl8 and cl5 as compared to the MEF GFP control. (**d**) TPC1 Matrigel migration analysis. Control plasmid-transfected TPC1 cells demonstrated higher migration levels than SOD3-transfected cells. (**e**) The intensity measurements of the migration images show a significantly (*p* < 0.001) decreased migration in SOD3 chambers. (**f**) Putative schematic presentation of the signaling pathways regulated by SOD3. The *p*-values are * *p* < 0.05 and *** *p* < 0.001.

Next, to qualify the protoarrays, we performed a Western blot analysis for the selected kinases (Figure 5a). Ephrin A2 phosphorylation was downregulated in MEF SOD3 cl5, thereby supporting the receptor array data (Figure 3a and Supplemental Figure S3). According to the previous data, Ephrin A2 mutant cancer cells demonstrated a decreased migration capacity, consequently resulting in a reduction in the number of metastasis in animal models [27]. The SRC oncogene; cJUN, AKT, and PKA substrates; and CREB demonstrated strong activation in MEF SOD3 cl5. TGFβ signaling is closely involved in tumorigenesis, demonstrating a growth inhibitor function at the early phase of tumorigenesis and growth promoter function at the late phase of carcinogenesis in cells that have a *TP53* mutation [28]. Therefore, we studied the mRNA expression of the *tgfβ1* and *tgfβ2* in MEF clones (Figure 5b). Both genes were downregulated by *sod3*, most apparently in SOD3 MEF cl5. The Western blot analysis for the phosphorylation of SMAD2/3 supported the mRNA expression data, suggesting a decreased activation of SMAD2 and SMAD3 in MEF SOD3 cl8 and cl5 (Figure 5c). To support the signaling data, we tested the effect of SOD3 on cancer cell migration using TPC1 papillary thyroid cancer cells. The Matrigel migration assay demonstrated a significant (*p* < 0.001) downregulation of cancer cell locomotion by SOD3 (Figure 5d).

In conclusion, the results in the present work suggest that an increased expression of SOD3, although causing DNA damage, did not induce a significant amount of point mutations or long-term microsatellite alterations that would affect the STR pattern used to identify the cell type. Therefore, we conclude that SOD3, or the reaction end product H_2_O_2_, are not mutagens. The data additionally demonstrated the activation of a large network of cancer-related signaling molecules by *sod3* overexpression (Figure 5f), corroborating the previous reports suggesting the growth regulatory nature of the enzyme.

## 4. Discussion

The availability of oxygen reconciles the production of ROS, which then engage in intra- and intercellular messaging. ROS are important signaling regulators targeting cysteine redox switches in proteins, modifying gene expression profiles, and even affecting cellular metabolic activity [29]. H_2_O_2_, besides being the most well-established ROS and a key player in oxidative stress, has a crucial role as a versatile secondary messenger regulating the growth and survival [9]. Within this concept, the function of the dismutase reaction end product, H_2_O_2_, synthesized at the cell membranes has not been thoroughly investigated yet. We have demonstrated previously that the forced expression of *sod3* in MEFs induces similar growth modalities as oncogenes—an initial proliferative burst followed by growth arrest and immortalization of the cells [30]. Oncogene-induced senescence (OIS), characterized by these three phases, followed by a subsequent escape of the cells from premature senescence, employ ROS as a downstream factor needed both for growth arrest and immortalization of the primary cells [31,32].

Although SOD3, or H_2_O_2_ produced by the enzyme, is not a growth factor, it can modify RAS GTP loading [22], hence adjusting the downstream growth stimulus. The enzyme has been shown to bind to caveolin-enriched lipid rafts, which provide a signaling platform for cell membrane-bound receptors, thereby regulating downstream kinases affecting, e.g., redox signaling [33,34]. Concomitantly, SOD3 synthesis is promoted by ERK1/2, forming a positive RAS-ERK1/2 feedback loop that could explain the increased cell proliferation and even the elevated healing process observed in tissue injuries. The imbalance in H_2_O_2_ production and too-high production of the ROS results in reduced growth, damage in cellular structures, and apoptosis. In the current work, we demonstrated that an increased dose of lentivirus SOD3 directly correlated with reduced MSC growth (Figure 1a), which could be a result of the fine-tuning of RAS GTPase activity [18] or DNA damage-induced activation of the DDR pathway (Figure 1b,f) [21].

The activation of p53 interrupts the cell cycle, allowing DNA damage repair enzymes to correct the DNA that subsequently abolishes the DDR signal [35]. Intriguingly, the SOD3 induced phosphorylation of p53 in two MEF clones, cl6 and cl5, with the concomitant downregulation of p21 and increased DNA replication as compared to GFP control cells (Figure 1f,g). SOD3, by synthesizing H_2_O_2_, induces cell growth and thereby increases the number of DNA replication forks during the initial proliferative burst (Figure 1a). Characteristically, during the intensive growth burst, the cellular mechanisms do not support all replication forks, which aim for cell division. The collapse of the replication forks causes DNA damage, with a subsequent increase in the DNA damage enzyme correction expression and DDR, as observed in Figure 1b,h. Therefore, the initial proliferative burst and subsequent collapse of the replication forks could form a plausible explanation for the increased p53 activation, but the mechanism for reduced p21 expression remains elusive. According to previous studies, the radiation damage in cancer cells can induce p53 activation, with the consequent binding of p53 to the distal promoter region of *p21* gene, thereby activating the transcription [36]. However, the expression of p21 is also stimulated by other signaling pathways, such as the TGFβ cascade, that initiates SMAD2/3 phosphorylation [37]. According to the current data (Figure 5b), the TGFβ ligand expression, especially *tgfβ1*, was produced at significantly lower levels in MEF SOD3 cl6 and cl5 as compared to the MEF GFP control cells. Other *p21* gene expression regulators include SP1, which, similar to p53 and TGFβ-SMAD-stimulated AP1, bind to the CIS-actin region of the *p21* gene [37].

Biological processes require energy in the form of ATP, which depends on the availability of oxygen, the primary source of ROS. In an aerobic organism, the ROS production is intimately linked to the mitochondria energy production chain and, therefore, to the cellular metabolism [1,38]. A balance of reduction–oxidation processes within an individual supports normal cellular metabolism, whereas oxidative stress, an increase of certain ROS molecules, are catalysts of the pathological processes. In support of this, a myriad of biological metabolic processes, such as hypoxia, inflammation, autophagy, proliferation, stem cell renewal, and aging, have a component of redox control [39]. In the current study, the gene ontology enrichment analysis data showed a large diversity of the biological networks influenced by SOD3, such as nitrogen and nucleic acid metabolic processes, suggesting SOD3 regulates amino acid, DNA, and RNA synthesis (Supplemental Figure S2). Metabolomics, consecutively, is regulated by the kinome, a kinase part of the proteome, in which the activation status varies between individuals, pathologies, and even different stages of a pathology [40,41].

Redox balance is a result of a dialog of different kinases that coordinate redox enzyme activation. The stimulus activating the kinase network and, therefore, restoration of the redox balance, can initiate from RTKs involving small GPTases or from GPCRs committing large G proteins [2]. An intriguing feature is that SOD3, which efficiently immortalizes the primary murine cells, intertwine the signaling routes commonly contributing to carcinogenesis, such as EphA/B, demonstrated in the current study (Figure 3 and Figure 5d). The function of the Eph receptor family has been studied in several cancers in which different Eph receptors, depending on the availability of the ligand and interaction with other RTKs, can act as tumor promoters or suppressors. In certain cases, they may even have a dual role in carcinogenesis [27,42]. The current data enforced the previous results, suggesting reduced cell migration caused by SOD3 (Figure 5d). It has been shown that the enzyme inhibits inflammatory cell migration to tissue damage by attenuating the expression of inflammatory cytokines and adhesion molecules at the injury site [42]. However, SOD3-derived inhibition of the locomotion of the cells that express SOD3 has not been studied thoroughly. Our current data suggested a correlation between the inhibition of cell migration and reduced SOD3-driven phosphorylation of EphA2, which promoted cancer cell migration, but the exact mechanism explaining SOD3 function in cell migration remains elusive.

Lastly, we compared the kinases network activated by SOD3 and SV40 in mouse embryonic cell-derived MEF and NIH3T3 cells. Since SOD3-driven signaling leading to murine cell immortalization differs markedly from immortalization promoted by the SV40 virus, which has the capacity to initiate tumorigenesis in mice, the comparison of the two could reveal novel biomarkers involved in the initiation of unwanted growth.

## 5. Conclusions

Therefore, in line with our previous data [22], the seemingly nonspecific wide-range signaling network regulated by SOD3 occurs concurrently with enforced cellular growth, which may include a cluster of kinases required for the initiation of tumorigenesis. Further analyses of the concomitant role of these cluster molecules in benign growth could potentially reveal modalities to prevent benign to malignant transformations of tumors.

## Data Availability

Not applicable.

## Supplementary Materials

The following are available online at https://www.mdpi.com/article/10.3390/antiox10050635/s1: Figure S1: Gorilla analysis. Biological process pathways affected by SOD3 in TPC1 cells modeling papillary thyroid cancer. Figure S2: Gorilla analysis. Biological process pathways affected by SOD3 in 8505c cells modeling anaplastic thyroid cancer. Figure S3: RTK array. (a) Protoarray long exposure for MEF GFP and MEF SOD3 cl5. (b) The locations of the signaling molecules on the membrane. Figure S4: RTK array. (a) Protoarray long exposure for MEF GFP, MEF SOD3 cl5, and NIH3T3 cells. (b) The location of the signaling molecules on the membrane. (c) Table showing the phosphorylation site in the kinases. Table S1: PCR primers that were used in the expression analysis.
