# Supplementary material for: SOD3 Is a Non-Mutagenic Growth Regulator Affecting Cell Migration and Proliferation Signal Transduction
Source: Antioxidants (Basel). 2021 Apr 21;10(5):635. doi: 10.3390/antiox10050635 (PMC8143115; doi:10.3390/antiox10050635)
Supplement: Supplementary file 1 [file antioxidants-10-00635-s001.zip › antioxidants-1178099-supplementary.pdf]

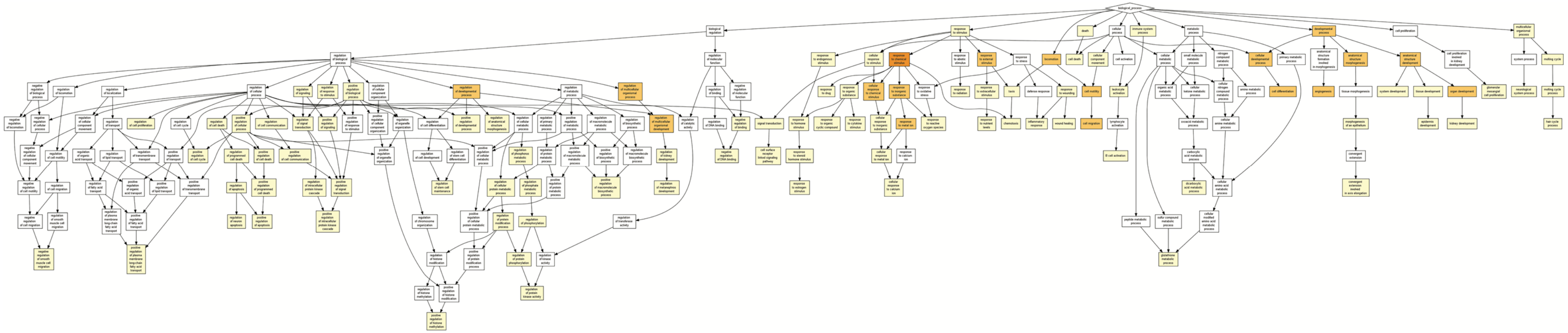

**Figure S1. Gorilla analysis**. Biological process pathways affected by SOD3 in TPC1 cells modeling papillary thyroid cancer.

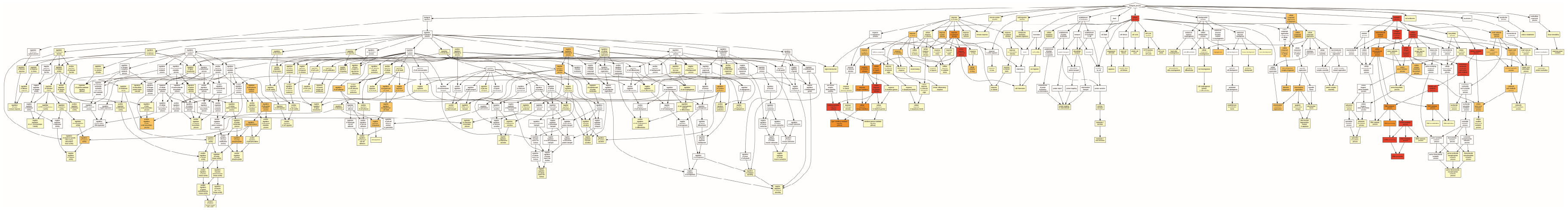

**Figure S2. Gorilla analysis**. Biological process pathways affected by SOD3 in 8505c cells modeling anaplastic thyroid cancer.

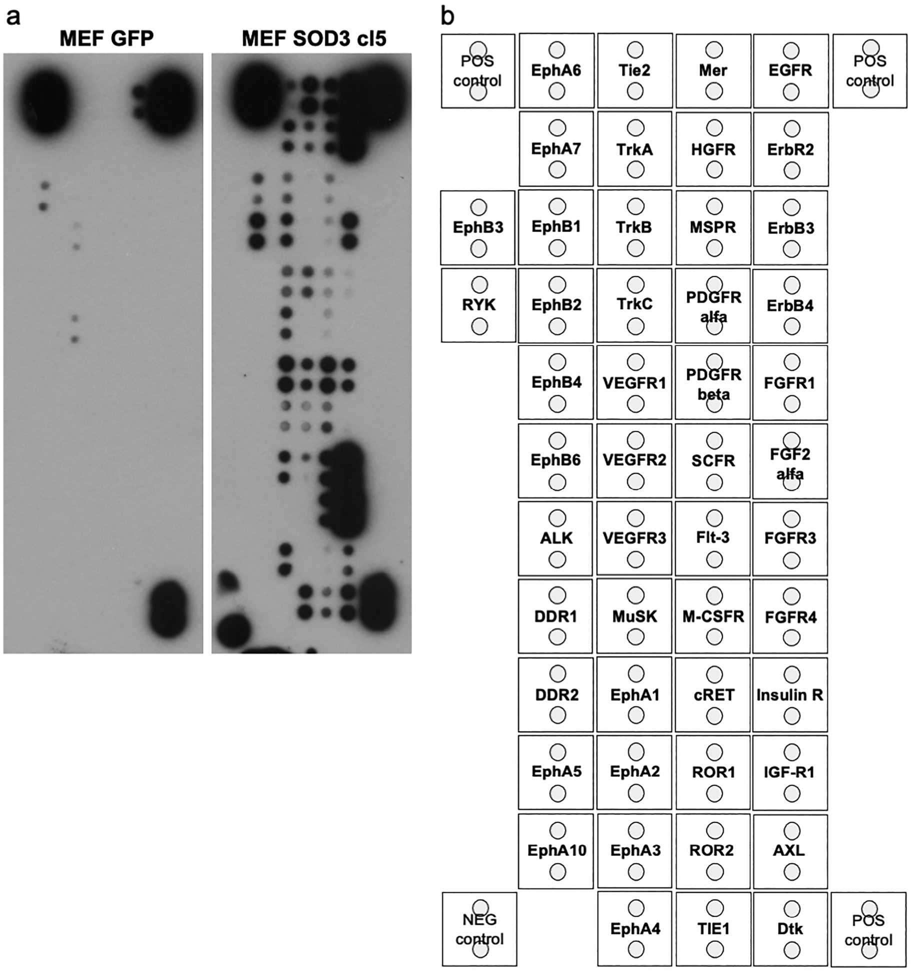


**Figure S3. RTK array**. a. Protoarray long exposure for MEF GFP and MEF SOD3 cl5. b. The locations of the signaling molecules on the membrane.

a b

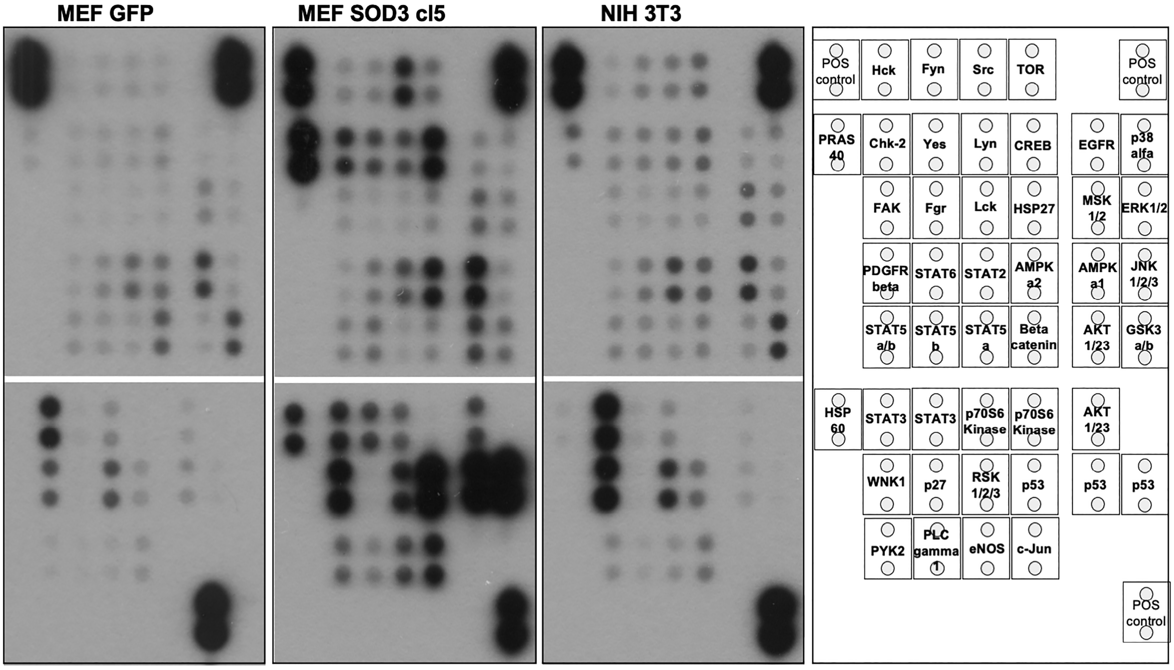


c

| | | | | | | |
|---|---|---|---|---|---|---|
| POS control | Hck Y411 | Fyn Y420 | Src Y419 | TOR S2448 | | POS control |
| PRAS40 T246 | Chk-2 T68 | Yes Y426 | Lyn Y397 | CREB S133 | EGFR Y1086 | p38 T180/Y182 |
| | FAK Y397 | Fgr Y412 | Lck Y394 | HSP27 S78/S82 | MSK1/2 S376/S360 | ERK1/2 T202/Y204,T185/ T185 |
| | PDGFR Beta Y751 | STAT6 Y641 | STAT2 Y689 | AMPKa2 T172 | AMPKa1 T183 | JNK T183/Y185/T221/ Y223 |
| | STAT5a/b Y694/Y699 | STAT 5b Y699 | STAT5a Y694 | Beta catenin | AKT1/2/3 S473 | GSK-3a/b S21/S9 |
| HSP60 | STAT3 S727 | STAT3 Y705 | p70S6 Kinase T421/S424 | p70S6 Kinase T389 | AKT1/2/3 T308 | |
| | WNK1 T60 | P27 T198 | RSK1/2/3 S380/S386/S377 | P53 S15 | p53 S46 | p53 S392 |
| | PYK2 Y402 | PLC gamma1 Y783 | eNOS S1177 | c-Jun S63 | | |

**Figure S4. RTK array**. a. Protoarray long exposure for MEF GFP, MEF SOD3 cl5, and NIH3T3 cells. b. The location of the signaling molecules on the membrane. c. Table showing the phosphorylation site in the kinases.

**Table S1.** PCR primers that were used in the expression analysis.

| | |
|---|---|
| **huDNA lig-I For** | GCT TCC ACC GAT TCC TCC TC |
| **huDNA lig-I Rev** | GAG CAC ACT GGG AAA ATG CC |
| **huDNA lig-III For** | GCC GGA GAG GCA GCT ATA TG |
| **huDNA lig-III Rev** | CAC GCC AGT GAT GTT TTC GG |
| **huMPG For** | GCC CAT ACC GCA GCA TCT AT |
| **huMPG Rev** | CCT CGG AGT TCT GTG CCA TT |
| **huUNG For** | CTC ATA AGG AGC GAG GCT GG |
| **huUNG Rev** | AGG GAT GAG CCG TCT GTA GT |
| **huOGG1 For** | AGG AAA GCC GGA GAA TTG GG |
| **huOGG1 Rev** | GAC GAG GAC CAC GAC CCT TA |
| **huNTH1 For** | GGA CAG TGA GAA AGG TGA GGC T |
| **huNTH1 Rev** | GTC CAC AGG TGC ATC CTT TTT G |
| **huNEIL1 For** | GTC ACT CAG TGC CCA AGG AA |
| **huNEIL1 Rev** | CCT GGA ACC AGA TGG TAC GG |
| **huNEIL2 For** | ATG GGT TAC AGA GGC TCA CCT |
| **huNEIL2 Rev** | AGA CGT CAG GTT CCC CAA CG |
| **huNEIL3 For** | TGG AAG TGC AGC TCA CCA AA |
| **huNEIL3 Rev** | AGC ACA TCA CCT AGC ATC CG |
| **huTDG For** | CTC TGG GGT TGT CTT ACC GC |
| **huTDG Rev** | CTG AAG GGA ATA GCT GCC CG |
| **huMBD4 For** | GCG CTT TCG CAA CAT TCA GA |
| **huMBD4 Rev** | CCC CAG ACT CAG ACT CTC CA |
| **huSMUG1 For** | TAA ACG ACC AGT GCT GGG AC |
| **huSMUG1 Rev** | AGC CAG GTC TTC TGA CTT GC |
| **huACTIN For** | TGC GTG ACA TTA AGG AGA AG |
| **huACTIN Rev** | GCT CGT AGC TCT TCT CCA |
